# Development of an Electronic Decision Aid Tool to Facilitate Mainstream Genetic Testing in Ovarian Cancer Patients

**DOI:** 10.1093/oncolo/oyad328

**Published:** 2024-01-31

**Authors:** Kristen M Shannon, Devanshi Patel, Jessica M Jonas, Erica L Blouch, Stephanie R Hicks, Mackenzie Wooters, Meredith Seidel, Carly F Grant, Margaret M Emmet, Daniel C Chung, Karen Sepucha

**Affiliations:** Massachusetts General Hospital Center for Cancer Risk Assessment, Boston, MA, USA; Massachusetts General Hospital Center for Cancer Risk Assessment, Boston, MA, USA; Massachusetts General Hospital Center for Cancer Risk Assessment, Boston, MA, USA; Massachusetts General Hospital Center for Cancer Risk Assessment, Boston, MA, USA; Massachusetts General Hospital Center for Cancer Risk Assessment, Boston, MA, USA; Massachusetts General Hospital Center for Cancer Risk Assessment, Boston, MA, USA; Massachusetts General Hospital Center for Cancer Risk Assessment, Boston, MA, USA; Massachusetts General Hospital Center for Cancer Risk Assessment, Boston, MA, USA; Massachusetts General Hospital Center for Cancer Risk Assessment, Boston, MA, USA; Massachusetts General Hospital Center for Cancer Risk Assessment, Boston, MA, USA; Division of Gastroenterology, Massachusetts General Hospital, Boston, MA, USA; Health Decision Sciences Center, Massachusetts General Hospital, Harvard Medical School, Boston, MA, USA

**Keywords:** genetic testing, ovarian cancer, decision aid, genetic counseling, shared decision-making

## Abstract

**Background:**

Multigene panel testing is an important component of cancer treatment plans and risk assessment, but there are many different panel options and choosing the most appropriate panel can be challenging for health care providers and patients. Electronic tools have been proposed to help patients make informed decisions about which gene panel to choose by considering their preferences and priorities.

**Materials and Methods:**

An electronic decision aid (DA) tool was developed in line with the International Patient Decision Aids Standards collaboration. The multidisciplinary project team collaborated with an external health care communications agency and the MGH Cancer Center Patient and Family Advisory Council (PFAC) to develop the DA. Surveys of genetic counselors and patients were used to scope the content, and alpha testing was used to refine the design and content.

**Results:**

Surveys of genetic counselors (*n* = 12) and patients (*n* = 228) identified common themes in discussing panel size and strategies for helping patients decide between panels and in identifying confusing terms for patients and distribution of patients’ choices. The DA, organized into 2 major sections, provides educational text, graphics, and videos to guide patients through the decision-making process. Alpha testing feedback from the PFAC (*n* = 4), genetic counselors (*n* = 3) and a group of lay people (*n* = 8) identified areas to improve navigation, simplify wording, and improve layout.

**Conclusion:**

The DA developed in this study has the potential to facilitate informed decision-making by patients regarding cancer genetic testing. The distinctive feature of this DA is that it addresses the specific question of which multigene panel may be most suitable for the patient. Its acceptability and effectiveness will be evaluated in future studies.

## Teaser

This article reports an electronic decision aid tool that was developed to help patients considering germline genetic testing make informed decisions about which gene panel to choose by considering their preferences and priorities.

**Implications for Practice**

The use of multigene panel testing in oncology has increased significantly, but selecting the most appropriate panel for each patient can be challenging for health care providers and patients. The complexity and heterogeneity of the data generated by these tests, coupled with the potential psychological, emotional, and financial impacts, make informed consent a critical component of the genetic testing process. With the expansion of germline genetic testing, the traditional model of pretest informed consent by a genetic counselor may no longer be scalable. Electronic decision aids have shown promise in increasing patient knowledge and empowerment in the shared decision-making process. The development of a comprehensive electronic tool can facilitate patient education while maximizing patient autonomy, allowing for more personalized and informed decisions about which gene panel to choose.

## Introduction

Germline genetic testing has become a critical component of oncology treatment plans for many patients, including those with breast, ovarian, colon, pancreas, and other solid tumors. One of the most widely used types of genetic testing is multigene panel testing, which can simultaneously analyze multiple genes associated with a particular disease or a group of related conditions. While multigene panel testing can provide important diagnostic and therapeutic information, choosing the most appropriate panel for each patient can be a challenging task for health care providers and patients. This is because different tests may cover different sets of genes, have varying sensitivity and specificity, and offer various levels of clinical utility, among other factors. As a result, the selection of the optimal test can be a complex and daunting task, which requires careful consideration of multiple clinical and genetic factors.

Multigene panel testing can generate a large amount of complex and heterogeneous data, which can be difficult for health care providers and patients to interpret and utilize effectively. In addition, the choice of a specific multigene panel test can have important implications for patients, including psychological and emotional impacts, and potential risks and benefits associated with the test. The variety of multigene cancer panel options complicates the process of informed consent as it relates to patient autonomy in medical decision-making.^1^ This critical element of the genetic testing process allows the patient to decide which, if any, multigene panel they would like to pursue, as aligned with their values. Genetic counselors typically invest a significant amount of time facilitating the detailed educational component and decision-making process for each patient considering multigene panel testing.

The clinical indications for germline genetic testing are expanding at a rapid pace, and germline genetic testing for all patients with solid tumors may soon be a reality.^2^ The traditional model of pretest informed consent performed by a genetic counselor is not scalable given the limitations in the genetic counseling workforce.^3^ Mainstream genetic testing is becoming an accepted alternative model, in which patients undergo pretest genetic counseling and informed consent by their nongenetics health care provider.^4^

Mainstream genetic testing has emerged as a transformative tool in oncology, facilitating rapid, cost-effective, and patient-centered diagnostics. This approach significantly influences the management of diseases like ovarian cancer. Several studies have demonstrated the effectiveness of mainstream genetic testing in improving patient outcomes by providing wider access to rapid, robust, and patient-centered germline genetic testing.^5–7^ Stearnes et al^8^ provided additional insights into the increasing uptake of testing with this mainstream genetic testing approach, emphasizing the growing awareness and acceptance of genetic testing among individuals and health care providers alike. Timely genetic testing aids in personalized risk assessment, enabling clinicians to make informed decisions about treatment strategies, prevention, and surveillance. Moreover, this approach empowers patients with knowledge about their genetic predisposition, fostering proactive health care decision-making, and enabling timely interventions to mitigate risks. The current literature underscores the importance of mainstream genetic testing in ovarian cancer patients, showcasing its positive impact on patient care and emphasizing its significance in shaping the future of precision medicine.

Electronic patient-facing educational tools have been developed to facilitate genetic testing and have been shown to increase patient knowledge and empowerment surrounding decision-making.^9^ Furthermore, studies show that clinicians using decision aids (DAs) report improved quality of care and satisfaction in the shared decision-making process.^10^ However, most tools address the decision to pursue one genetic testing option rather than which multigene panel to select among the many options. Given the promising preliminary data, we proposed the development of a comprehensive electronic tool for ovarian cancer patients to facilitate patient education while maximizing patient autonomy. Our goal was to have patients select one of 4 options: pursue a smaller panel of clinically actionable genes related to ovarian cancer, pursue a medium-sized panel of mostly clinically actionable genes across cancer types, pursue a large panel of both clinically actionable and nonactionable genes across cancer types, or decline genetic testing. Here we describe the design of this electronic DA tool designed to help patients to make more informed and personalized decisions about which gene panel to choose by considering their preferences, values, and priorities.

## Methods

### Rationale for DA

Massachusetts General Hospital (MGH) is a large academic medical center in Boston, Massachusetts, part of the Mass General Brigham healthcare system. As a comprehensive cancer center, MGH Cancer Center provides expert care for patients with diverse types of cancer, including ovarian cancer. Most patients with ovarian cancer are treated by highly specialized gynecologic oncologists. The MGH Center for Cancer Risk Assessment (CCRA) provides genetic counseling and testing services to a wide variety of patients with and without cancer. Despite a staff of 14 genetic counselors, the number of cancer patients requiring access to genetic testing was outpacing the clinical capacity. Thus, we explored the possibility of mainstreaming genetic testing in ovarian cancer patients, meaning the patient would be educated and consented to genetic testing by the treating gynecologic medical oncologist.

When we proposed a mainstream genetic testing model to the gynecologic oncology providers at our institution, they identified concerns with both the pretest informed consent process and the post-test follow-up. Specifically, they were concerned about the long-term monitoring of variants of uncertain significance (VUS). Genetic counselors were readily available to assist patients and health care providers with any questions or uncertainties related to VUS results. This approach ensured that the testing process was not only patient-friendly and informative but also encompassed robust support mechanisms for result disclosure and ongoing monitoring of genetic variants.

Providers also identified 2 main concerns with the pretest counseling associated with the mainstream approach. They expressed concern for a lack of expertise in providing patient education and in helping patients choose the most appropriate gene panel. Their bigger concern, however, was the amount of additional clinic visit time required given the lengthy nature of the informed consent process. We sought to alleviate these 2 concerns by developing a patient-friendly intervention to assist in the patient education and decision-making process.

### Multistep Framework

Our DA was developed in line with the framework described by Coulter et al^11^ and put forth by the International Patient Decision Aids Standards (IPDAS) collaboration, which consists of 5 steps: (1) defining the scope, (2) design, (3) prototype development, (4) alpha testing and steering committee review, and (5) beta testing. Here we focus on the first 4 steps; beta testing is occurring in both a pilot study and as a randomized controlled trial and will be reported separately.

#### Defining the Scope

Our DA focused on a target audience of ovarian cancer patients for 3 main reasons. First, germline genetic testing for ovarian cancer patients is universally recommended,^12^ obviating the need to determine eligibility for testing based on family history or other factors. Second, because of the universal recommendation, most insurance companies cover the cost of germline genetic testing for ovary patients, mitigating the discussion of out-of-pocket cost. Finally, ovarian cancer is a relatively uncommon diagnosis, with <100 new patients diagnosed at our institution each year, making this an ideal population in which to pilot a mainstream genetic testing model.

**Figure 1.**
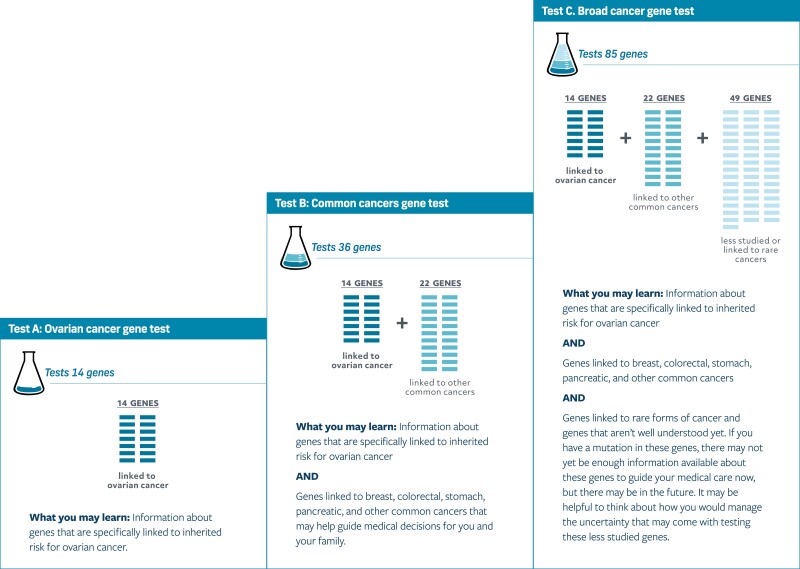
Gene panel testing options.

Our project team was comprised of genetic counselors, medical oncologists, decision scientist, and health communication experts. As is consistent with our current clinical genetic counseling practice, the team wanted a DA that provided multiple options for genetic testing to be offered to patients. Throughout, we wanted the DA to use a combination of written information, graphics, and audio/video content to accommodate various learning styles. We planned to include patient quotes as part of the educational strategy. Finally, following IPDAS, we wanted to include a values clarification exercise and a summary component to assist patients in decision-making regarding 3 panel options for genetic testing (Fig. 1).

After an extensive literature review, we found there were various genetic testing DAs available, but few were truly interactive or designed for patient use.^13,14^ In addition, most DAs for cancer predisposition testing were tailored to syndrome-specific/gene-specific testing.^15,16^ Our team reached out to the National Society of Genetic Counselors (www.nsgc.org), who expressed support for this unique project. We secured an educational grant from Astra Zeneca (Grant ID# 41473887) for the development of the electronic DA.

#### Design

Our project team collaborated with an external health care communications agency (Health Communication Core [HCC]) and the MGH Cancer Center Patient and Family Advisory Council (PFAC) to identify key information about the germline genetic testing and informed consent process that would be essential to patient understanding.

**Figure 2.**
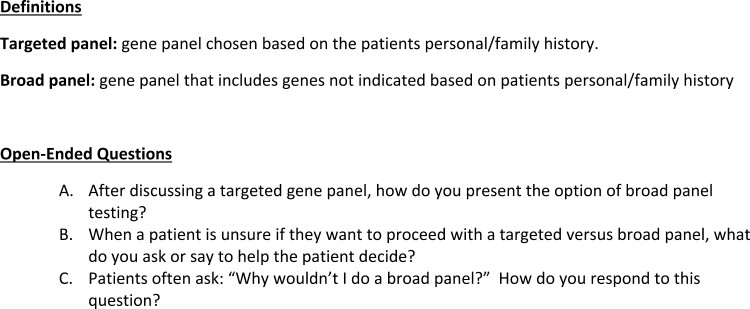
Current practice of genetic counselor survey.

The HCC held discussions with the project team to clarify how the DA would be integrated into the clinical flow and underwent mock counseling sessions with the CCRA genetic counselor staff to determine how best to replicate in-person counseling in the DA. Once the major educational topics were identified, we sought to determine the appropriate flow of the aid and decided to gather more information on current practice models. Our project team investigated how genetic counselors were facilitating patient decision-making in the traditional setting, and we conducted an online anonymous survey of the CCRA genetic counselors. Two terms were defined, and the 3 questions were asked in an open-ended text box (Fig. 2).

Finally, the Project team developed an IRB (Institutional Review Board) study (MGB IRB#2019P000342) to survey patients and genetic counselors to learn more about how individuals make decisions about genetic testing. The study aimed to describe the frequency with which patients choose one of several panel options or decline genetic testing and to identify variables that may predict patient choice.^17^ One goal of this survey project was to identify values-based assessments that could be used in the development of our DA.

#### Prototype Development

The design steps above resulted in the development of interactive educational content and values-clarification sections that reviewed and reinforced information while encouraging users to make a decision. Patient responses were compiled into a summary that patients could opt to have emailed to them before they exited the DA. Videos of genetic counselors explaining key concepts were produced by an outside vendor. Audio “testimonials” of patients discussing their decision-making process and representing diverse decisions were obtained. The HCC developed easy-to-understand graphics illustrating cancer risk and other genetic testing concepts. Users of the DA were able to move through the DA sequentially, access optional in-depth content, skip or return to specific sections, or jump to the “choose a genetic testing panel,” based on their personal preferences, level of knowledge, and readiness to decide. Users could also add questions and comments as they moved through the DA, which were then compiled in the downloadable summary.

#### Alpha Testing and Project Team Review

To refine the prototype, we conducted alpha testing with PFAC members and genetic counselor project members. Each PFAC member met individually with a member of HCC who used a semistructured interview guide to gather information on the readability, relevance, and acceptability of the DA as the PFAC member navigated through the tool. Each genetic counselor project member tested the DA and provided feedback on readability, relevance, and acceptability. In addition, we informally had 8 lay people navigate the DA and provide feedback on readability and acceptability. The iterative process of revising the DA was overseen by a multidisciplinary steering committee consisting of the project team and the HCC team.

## Results

### DA Design

**Figure 3.**
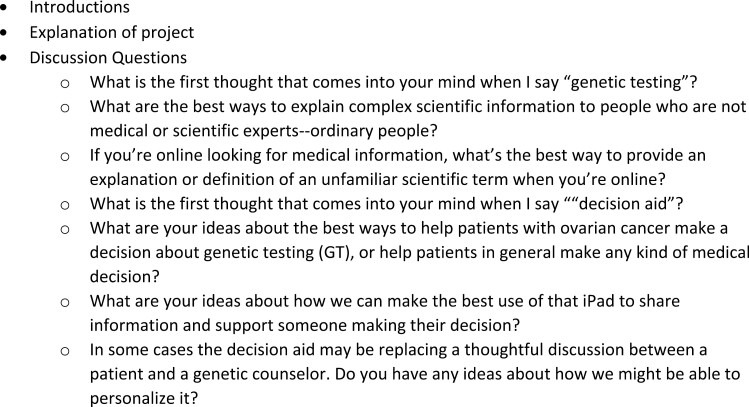
PFAC focus group discussion guide.

The HCC and project team held a one-hour focus group with 3 (female) members of the PFAC. After an introduction to the project, the PFAC members were asked general questions about genetic testing, how they accessed information online, and their thoughts on how to explain complex scientific information to lay people (Fig. 3). The focus group was audio recorded and transcribed. The PFAC contributions to the future DA development included ideas on readability, the ratio of text and video content, and key messages (eg, impact on family).

**Figure 4.**
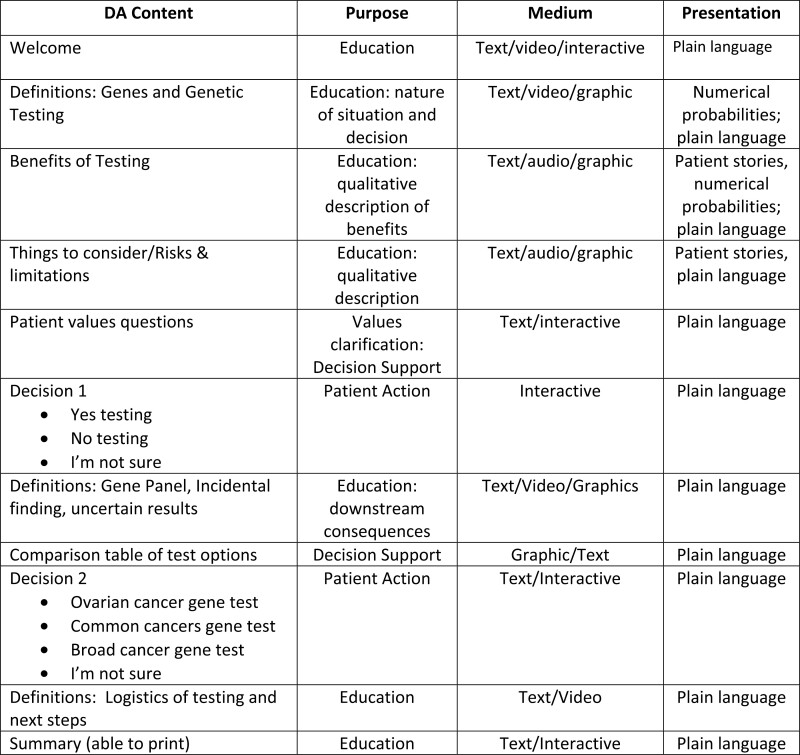
Electronic DA content and purpose of content.

The key educational content was ascertained from the genetic counselor team members. The content underwent 4 revisions by 3 genetic counselors on the project team before being sent to HCC for plain language support. The topics can be seen in Fig. 4 with brief descriptions of content type.

When surveying the genetic counselors regarding current practice issues, 12 CCRA genetic counselors responded to an anonymous survey. Common themes were identified and categorized. Most genetic counselors (10/12) started the discussion about panel size with a description of targeted genetic testing and then expanded to include broad genetic testing panel options. One genetic counselor reported discussing the broadest panel first and then “worked backward” to the targeted panel, and one genetic counselor discussed a broad panel as a reflex option in the context of a negative targeted panel. When asked for strategies for facilitating patient decision-making, genetic counselors most commonly explored how the patient dealt with uncertainty (8/12); how the patient felt about getting an unexpected but clinically relevant finding (5/12), and if the patient would describe themselves as an “information seeker” (4/12). Other strategies included exploring if the patient found medical information particularly anxiety provoking, if they were someone who needed to know the “latest and greatest,” if they could compartmentalize information well, and if they were comfortable with information changing over time. Finally, when asked “why wouldn’t I do a broad panel?,” genetic counselors most often cited 3 patient perspectives: 1. Some people did not like uncertainty (6/12); 2. Some patients were concerned about a higher rate of a VUS result in the broad panel (6/12); and 3. Some patients only wanted information relevant to their current circumstances (5/12). These genetic counselor survey responses informed the educational components in the DA (Fig. 3) and in some of the text used to describe the risks and benefits of different panel types.

Our patient survey study (Partners Healthcare IRB Protocol #2019P000342) was designed to explore patient choice between a full spectrum of multigene panel options.^17^ It examined factors associated with the selection of a specific multigene panel test by patients when undergoing pretest education and informed consent with a genetic counselor. The study confirmed the importance of patient choice, provided insight into the distribution of patients’ preferences for testing, and supported the idea that a DA may be helpful in facilitating informed decision-making.

### Prototype

The full tool is available at https://mghcancergeneticsda.com/. The project team organized the information into 2 major sections: 1) deciding about genetic testing (yes/no) and 2) deciding which panel type (ovarian cancer, common cancer, and broad cancer). Each major heading is followed by educational points that expand in an accordion style to provide additional context. Provider videos support the learning process and patient quotes are used to provide insight into the patient experience with genetic testing.

The learning process focuses first on the decision to have genetic testing (versus not having genetic testing) and includes value statements that the user rates on a 7-point Likert scale. The user is then prompted to decide if they would like to proceed with genetic testing. If the user decides they do not want genetic testing, they can either end the DA tool or continue to learn more about testing options. The user can also indicate they are not ready to decide about genetic testing. If this is the case, they are asked if they would like to speak with their oncologist, speak with a genetic counselor, or take some time to think about it. Finally, if the user decides to proceed with testing, they are guided through the second educational aspect of the DA: education and decision-making regarding which gene panel to pursue. Users are provided with the 3 testing options, including the limitations and benefits of each panel type (Fig. 1). Once the education is completed, the patient is prompted to choose one of 3 tests. The final education section of the tool provides testing logistics and next steps (eg, getting a blood draw and turnaround time of test). Once the DA is completed (whether a test is chosen), the user receives summative guidance about which decision they have made and can email it to themselves for their records.

### Alpha Testing

Four members of the PFAC assisted with the Alpha testing. Three of these (female) individuals had participated in the design focus group and one (male) was a new member. PFAC members suggested changes-based acceptability and readability. Changes to the DA because of the PFAC testing included adding a welcome screen at the outset of the DA, moving the navigation bar to the top of the screen, and creating additional accordion buttons to reduce the amount of text on a page. Changes to readability included removing redundant text and labeling statistical information more clearly.

The 8 lay people who navigated the DA agreed the content was relevant and readable, but suggested text modifications to reduce redundancy. Modifications were made to the navigation based on their feedback, including the addition of a “back” button to return to a previous page or section and a redesign of the accordion feature to make it more readable.

The genetic counselor project team members identified minor navigation concerns (eg, when clicking a button, it did not skip to the correct location) which were rectified with programming.

## Discussion

We describe the development of a DA for multigene panel cancer genetic testing that facilitates informed decision-making by patients. The distinctive feature of this DA is its ability to tackle the intricate task of facilitating the selection of the most suitable multigene panel test, a challenge that has been widely recognized in the medical community. The increasing number of available multigene panel tests has created a significant dilemma for clinicians, who struggle to identify the most appropriate option for each patient’s unique clinical scenario. This DA tool offers a comprehensive and personalized approach to guide clinicians toward a more informed and evidence-based decision-making process.

The educational content of the DA underwent several revisions, and patient surveys were conducted to explore factors associated with the selection of a specific multigene panel test. A patient survey study performed during the development process showed that while large panels were common, testing choice was distributed across different panel options and was difficult to predict.^17^ This highlights the importance of patient choice and the need for a DA to facilitate informed decision-making.

The alpha testing phase included feedback from Patient and Family Advisory Council (PFAC) members and genetic counselor project team members. PFAC members provided significant feedback resulting in major navigation and content changes. Minor navigation concerns were identified by the genetic counselors, and rectified, and lay people navigated the DA and suggested no changes. This feedback indicates that the DA is user-friendly and can be used by a wide range of patients.

The tool has certain limitations that should be noted. First, DAs may not be appropriate for all patients, particularly those with complex medical histories or comorbidities.^18^ Further, our DA is currently available in only one language and designed for a specific disease population, namely ovarian cancer. It is worth noting that plans are in place to address these limitations. Consistent with research by Lau et al,^19^ which highlights the importance of considering cultural and linguistic factors when developing DAs for diverse patient populations, we will be translating the tool into multiple languages. In addition, we will be broadening its applicability to other cancer types.

## Conclusion

The DA developed in this study has the potential to facilitate informed decision-making by patients regarding cancer genetic testing. The DA’s effectiveness and impact on patient decision-making will be evaluated in future studies to validate its utility.

## Data Availability

The data underlying this article will be shared on reasonable request to the corresponding author.
